# The Perception of Pharmacology Among College Students: An East London Perspective

**DOI:** 10.1002/prp2.70157

**Published:** 2025-07-28

**Authors:** Cara Camilla Chantry, Elisa Salinas Pérez, Sahil Seyal, Saarah Mohammed, Devyani Deshai, Kristine Ofori, Muhummed Awan, Rayna Haque, Florence Olivia Mehtar, Samir S. Ayoub

**Affiliations:** ^1^ School of Health, Sport and Bioscience, Medicines Research Group University of East London, Stratford Campus London UK

**Keywords:** curriculum, education, perception, pharmacology

## Abstract

Pharmacology is an integrative discipline that plays an integral part in the development of new medicines with improved safety and efficacy profiles. Sustained growth of this important discipline within the UK is made possible through training of the next generation of pharmacologists. In order to ensure that interest in pharmacology continues to grow, endeavors aimed at exposing students to pharmacology from earlier stages of their educational journeys have to be put in place. To this end, the current study aimed at capturing the perception of further education students on pharmacology in the East London area. This survey‐based study, which took place between 2020 and 2021, consisted of multiple choice questions. The study revealed that over 80% of the surveyed biology and chemistry students have previously heard about pharmacology. However, when assessing their basic knowledge of pharmacology, it emerged that students had a somewhat distorted perception of pharmacology, as only 9.8% of the students associated pharmacology with biology. Additionally, students confused pharmacology with pharmacy. Students also had a somewhat limited understanding of what pharmacologists do. Finally, 23.5% of the students stated that they would consider studying pharmacology at university if they received sufficient introduction, with 92.2% of the students stating that they would like to see pharmacology added to their further education curriculum. In order to ensure the growth of pharmacology in the UK and given the misconceptions that students have, as highlighted in this study, we recommend that basic pharmacology education be introduced to the further education curriculum.

## Graphical abstract

An overwhelming majority (over 90%) of students included in this survey study recommend that pharmacology education should be included in their further education curriculum.

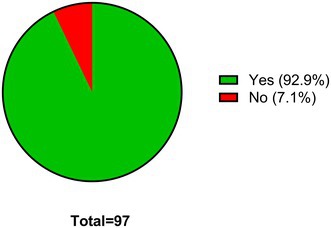

## 1 Introduction

Pharmacology is the scientific and research discipline that seeks to elucidate the pharmacological and toxicological actions of drugs and to determine the mechanisms through which these actions are mediated. Pharmacology plays an integral part in every step in the drug discovery and development process and in our understanding of the underlying disease processes at the molecular level. The development of effective and safe drugs is made possible through rigorous pharmacological research. Given the prevalence of chronic and debilitating diseases that include cancer, cardiovascular disease, and neurological disorders, it highlights the importance of pharmacology as a research discipline in the development of medicines for these conditions [1].

Given the prevalence of chronic human disease and the emergence of infectious disease outbreaks such as the COVID‐19 pandemic, it is important to ensure the continuity and growth of this discipline. It is therefore important to ensure that we continue to train the next generation of pharmacologists. Historically, the United Kingdom has played a central role in the development of pharmacology through the work of eminent scientists that include Alfred Joseph Clarke [2], Joshua Harold Burn [3], Sir William Paton [4], Sir John Gaddum [5], and Sir Henry Hallett Dale [6] and more recently through eminent pharmacologists that include Sir James Black [7] and Sir John Vane [8]. Their research and discoveries have paved the way to today's therapeutics.

Within the UK, pharmacology is offered as a Bachelor's degree at more than 40 universities as a single Honors degree or at some institutions as a combined Honors degree and with or without the option of a 1‐year industrial placement. Some universities also offer pharmacology as a 4‐year Masters degree. Despite the widespread provision of pharmacology education within the UK and the varied recruitment of students into these courses [9], there still seems to be many myths and misconceptions around pharmacology among the pre‐university student population. Such misconceptions typically include the confusion between pharmacology and pharmacy. It is therefore important to generate more awareness and to dispel such misconceptions among the further education students, while also attempting to establish why students may or may not be attracted to pharmacology. In order for that to happen, it is important to capture young people's awareness and perceptions on pharmacology. As there is no evidence of such research having been conducted, the current study sought to establish the perceptions of further education students of pharmacology and has used the east London area as a model due to the close geographical proximity to the authors' institution. Some interesting trends have emerged from this survey study, which warrant further attention.

## 2 Methods

This study was conducted in the form of a survey, which was granted ethical approval by the University of East London Research and Ethics Committee in April 2019 (Approval No. ETH1819‐0143). The survey was completely anonymous as no personal information was collected. Consents were obtained from participants (students) and their parents and carers (See Appendices S1 and S2 for templates of the consent forms) prior to commencement of the surveys. Along with the consent forms, all involved individuals were provided with the Participants Information Sheet.

The survey questions, which were constructed in the form of multiple choice questions, is shown in Tables 1a and 1b. The survey was divided into two parts with the questions in the first part being aimed at capturing the students' perceptions of pharmacology. The students within the classroom setting were then shown the video entitled “Studying Pharmacology at University” (https://www.youtube.com/watch?v=efsKa37cU2M), which is a 2.21 min video that has been put together by the British Pharmacological Society. This video gives pre‐university students a brief introduction to pharmacology and what students can do with a degree in the pharmacology. The students were then given part two of the survey to complete, which aims to capture the students' opinions about the inclusion of some pharmacology education as part of their further education curriculum and whether they would consider studying pharmacology at university now that they have been informed on what pharmacology as a discipline aims to achieve.

**TABLE 1a:** Part one of the survey questions on the perceptions of further education students on pharmacology.

Question no.	Question	Options to choose from
1	Have you ever heard of *pharmacology*?	Yes No
2	If you have heard of pharmacology, where did you hear about it?	Universities course listing UEL course listing My school/college Career event General press Documentary British Pharmacological Society Friends/family Social media, please specify……………………………………. Other (please state)…………………………………………………. Have never heard of pharmacology
3a	State which one or more words from the list below you think are most relevant to *pharmacology*?	Pharmacy Chemistry Maths Drugs Plants Pharmaceutical industry Medicines Biology
3b	Qn3b. List the top three words from the list above that you think are mostly related to pharmacology (in no particular order).	Pharmacy Chemistry Maths Drugs Plants Pharmaceutical industry Medicines Biology
4	Select one or more statements from the list below that you think best describe what *pharmacology* is all about.	Manufacturing of new medicines Determining the side effects of new medicines Dispensing of medicines to patients Studying the mechanism of action of medicines on the body Determining the correct dose of medicine to give to patients Studying the effect of medicines on the body Design, packaging and assembling of medicine Marketing of medicines Nothing to do with medicines
5	State which one or more of the following statements describe what do you think *pharmacologists* do?	Develop new medicines with better therapeutic effects and less side effects Understand more about how the body works Improve government regulations on prescribing and selling medicines Understand more about how drugs work Develop medicines to treat diseases that have no current treatments Provide awareness to the general public on medicines, their benefits and risks Dispense medicines at pharmacies/hospitals Other, write your answer……………………………………………………………
6	Pharmacologists investigate the action of drugs at which one or more level?	Molecular Organ Cellular Biochemical Organism All of the above
7	A degree in pharmacology enables graduates to work only as *pharmacologists*.	True False

**TABLE 1b:** Part two of the survey questions on the perceptions of further education students on pharmacology.

Question no.	Question	Options to choose from
8	If you were taught about pharmacology as part of your current studies, would you consider studying it at university?	Yes No Maybe Have not thought about it
9	Do you think pharmacology should be introduced to secondary or further education curriculum?	Yes No

The survey questions were designed with the aim of assessing the students' level of knowledge about pharmacology. The first question was aimed at identifying from the student answers who have heard about pharmacology (Question 1). The next logical question was to find out the source through which they have come to hear about pharmacology (Question 2). Following on from that, it was important to assess the students' depth of knowledge and to capture their perceptions about pharmacology, as reflected in questions 3–7. Questions 3a and 3b were word association questions, for which the students were given the option of choosing words that are directly related to pharmacology, such as the word drug, or words that are indirectly related, such as the word plant, with the aim of finding out the depth of their knowledge about pharmacology. Hence, the more that students would choose words that are directly related to pharmacology, the better acquainted they would be with the subject. Questions 4–7 are aimed at further probing the students' perceptions and depth of knowledge about pharmacology, this time not by using words but by choosing statements that are again directly relevant, indirectly relevant, or not relevant to pharmacology. Part 2 of the survey was aimed at identifying whether the students have developed any interest in pharmacology after having watched the short video (Questions 8 and 9). Questions 1 and 7–9 required a single answer, whereas for Questions 2–6, students had a choice of several responses to choose from.

This study started in January 2020 and concluded in May 2021. Initially, the study took place in person with paper completion of the surveys. Following the COVID‐19 lockdown as of March 2020, the rest of the surveys were completed online with the use of Microsoft Forms. With the paper and online surveys, the students access to sources of information was strictly prohibited in order to ensure that their responses to the survey reflect their state of knowledge on pharmacology. Table 2 shows the information related to all survey completions at the different schools and colleges. In summary, a total of 97 students were surveyed from 3 colleges, of whom 86 were BTEC Level 3 Applied Science students and 11 were year 12 A Level Biology and Chemistry students. Two BTEC Level 3 Applied Science students from New City Redbridge were surveyed in consecutive academic years.

**TABLE 2:** Information related to each survey completion.

School	Date survey completed	Course name, subject(s) & level of study	No. of students	Onsite or online
London Academy of Excellence	February 7th, 2020	A level Biology and Chemistry, year 12	11	Onsite
New City College Redbridge	January 28th, 2020	BTEC Level 3 Applied Science	35	Onsite
Newham Sixth Form College	January 31st, 2020	BTEC Level 3 Applied Science	30	Onsite
New City College Redbridge	May 26th, 2021	BTEC Level 3 Applied Science	21	Online

## 3 Results

### 3.1 The Outcomes From Survey Questions Aimed at Capturing the Percentage of Students Who Know About Pharmacology and the Source of This Knowledge

**FIGURE 1:**
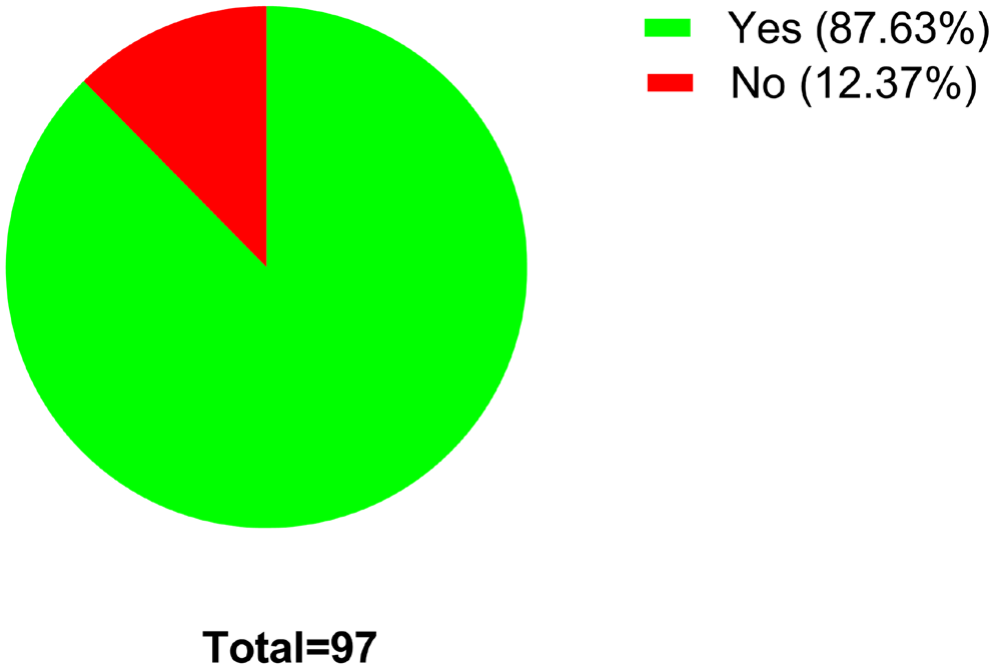
Have you ever heard of pharmacology? Students were requested to provide a single response for this survey question.

**FIGURE 2:**
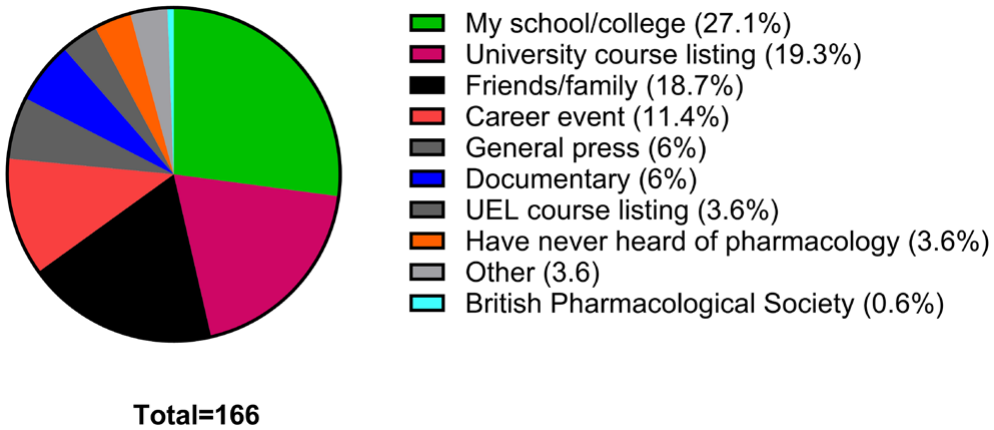
If you have heard of pharmacology, where did you hear about it? Students were expected to indicate all answers that applied.

The survey data presented here represent pooled data from all the students at all the colleges included in the study. The first question in the survey enquires about whether the students have ever heard about pharmacology. From the responses generated, 87.63% of all students (97 students in total) have stated that they have heard about pharmacology, with only 12.37% stating that they never heard about it (Figure 1). Of those who stated that they have heard about pharmacology before, 19.3% stated that they heard about pharmacology through university course listings (Figure 2). The students have also stated that they heard about pharmacology through their colleges (27.1%) and through friends or family members (18.7%).

### 3.2 The Outcomes From Survey Questions Aimed at Assessing the Depth of Students' Knowledge and Perception About Pharmacology

**FIGURE 3:**
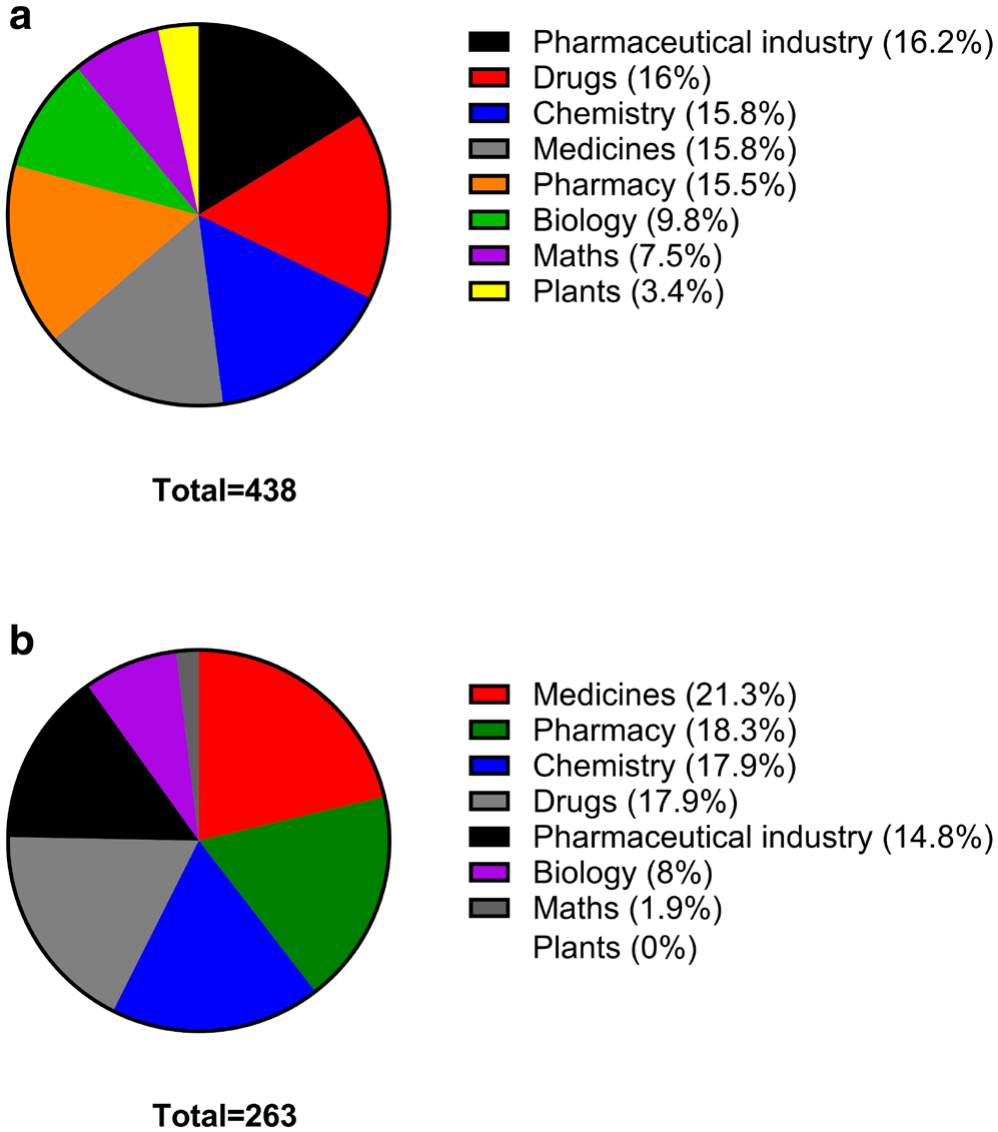
(a) State which one or more words from the list below you think are most relevant to pharmacology? Students were expected to indicate all answers that applied. (b) List the top three words from the list above that you think are mostly related to pharmacology (in no particular order). Students were expected to indicate all answers that applied.

Despite the high level of awareness on the existence of pharmacology as a scientific and medically‐related discipline by students, as demonstrated from the data presented in Figure 2, students seem to have a somewhat distorted perception about pharmacology, as only 9.8% of the students associated pharmacology with biology (Figure 3a). In Figure 3a the students were asked which one or more words they thought are most relevant to pharmacology and were asked to select from the following words: pharmacy, chemistry, maths, drugs, plants, pharmaceutical industry, medicines, and biology. In Figure 3b the students were then asked to select the top three words from the above list that they thought are mostly related to Pharmacology (in no particular order). It is quite interesting that, again, biology was ranked at 8% only (second lowest) much lower than chemistry (17.9%) (Figure 3b). Students quite often confuse pharmacology with pharmacy; it is therefore no surprise that students ranked pharmacy at 18.3% (second highest). That said, students were also able to correctly associate pharmacology with medicines (21.3%) and drugs (17.9%).

**FIGURE 4:**
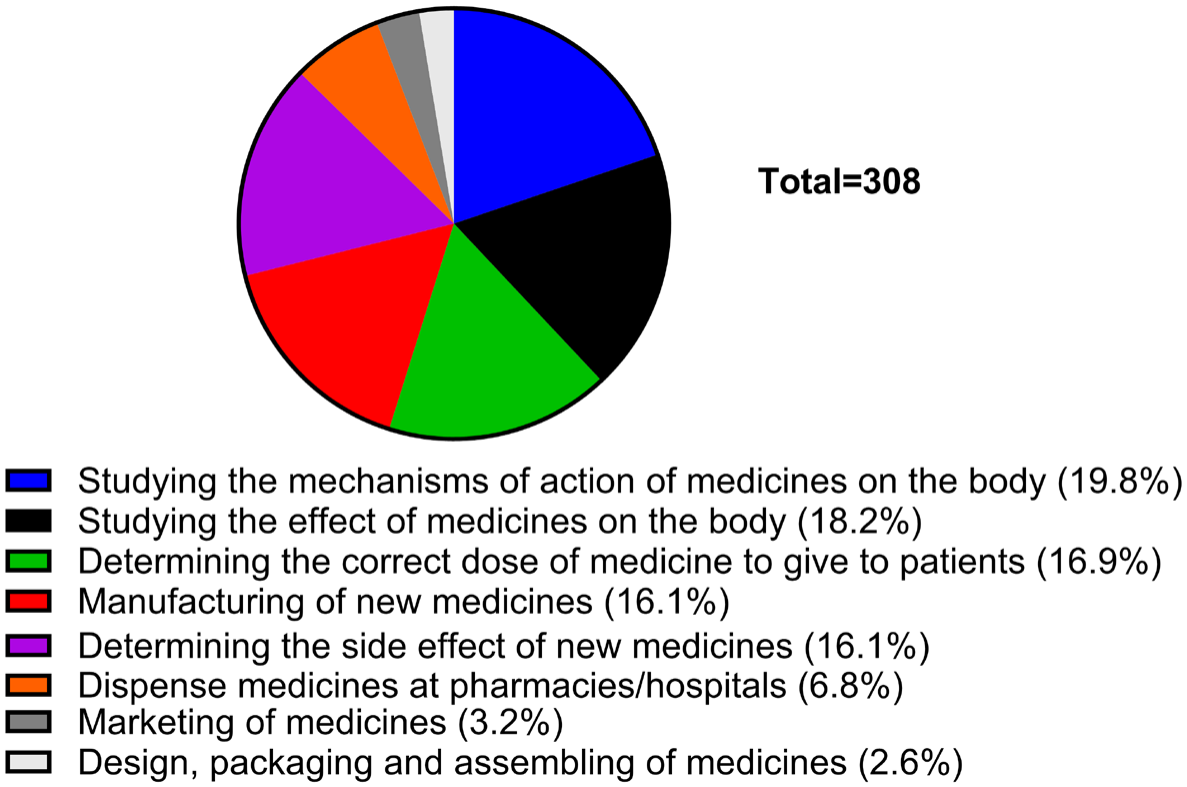
Select one or more statements from the list below that you think best describe what pharmacology is all about. Students were expected to indicate all answers that applied.

Next, in Question 4, students were asked to select one or more statements that they believe best describe what pharmacology is all about. Students seemed to mostly select the relevant statements, which included studying the mechanism of action of medicines on the body (19.8%), determining the correct dose of medicine to give to patients (16.9%), studying the effect of medicines on the body (18.2%), and determining the side effects of new medicines (16.1%); all of which are correctly attributable and directly relevant to pharmacology and were highly ranked by the students. That said, the students also attributed the following statements to pharmacology: manufacturing of new medicines (16.1%) and dispensing of medicines to patients (6.8%) (Figure 4).

**FIGURE 5:**
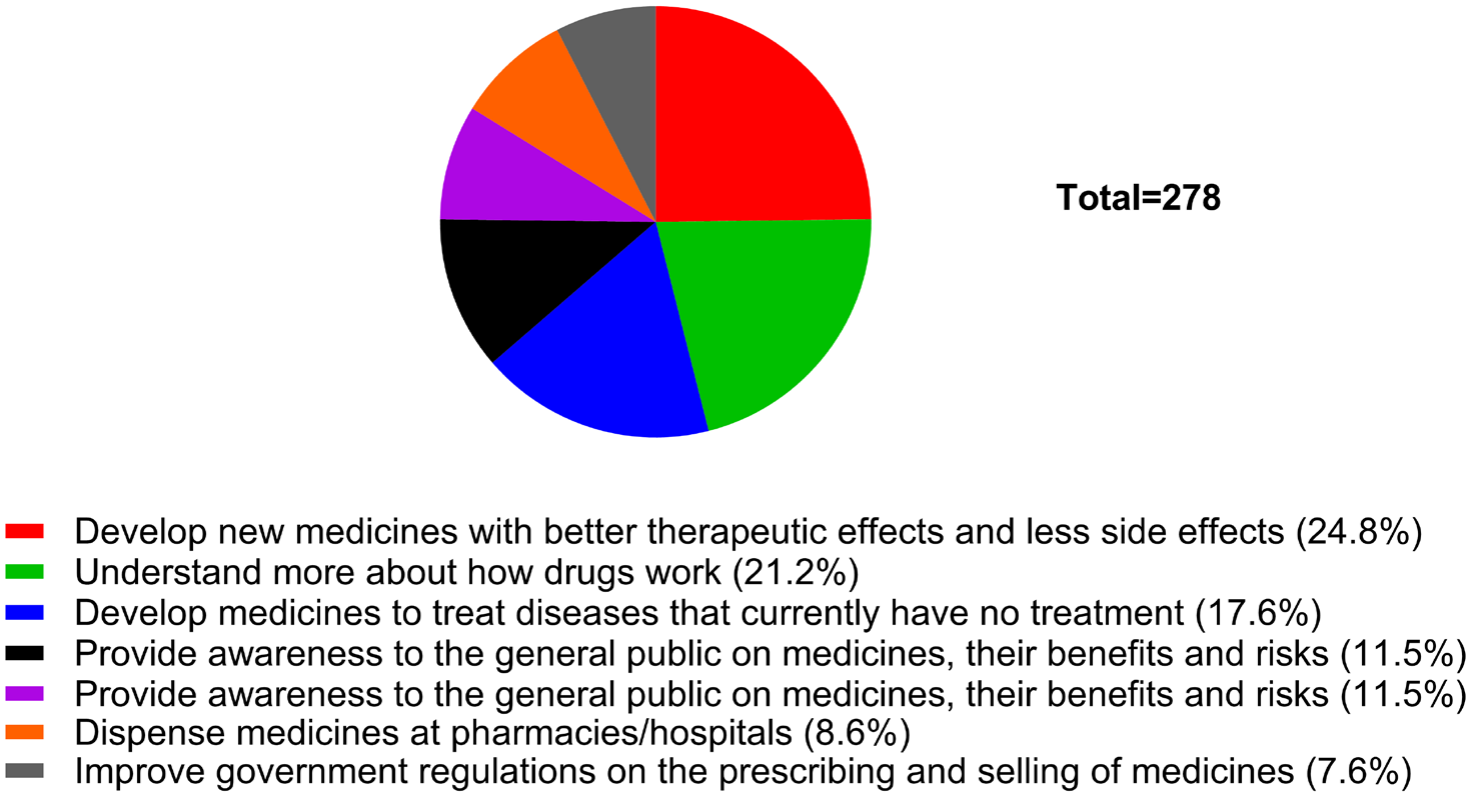
State which one or more of the following statements describe what do you think pharmacologists do? Students were expected to indicate all answers that applied.

**FIGURE 6:**
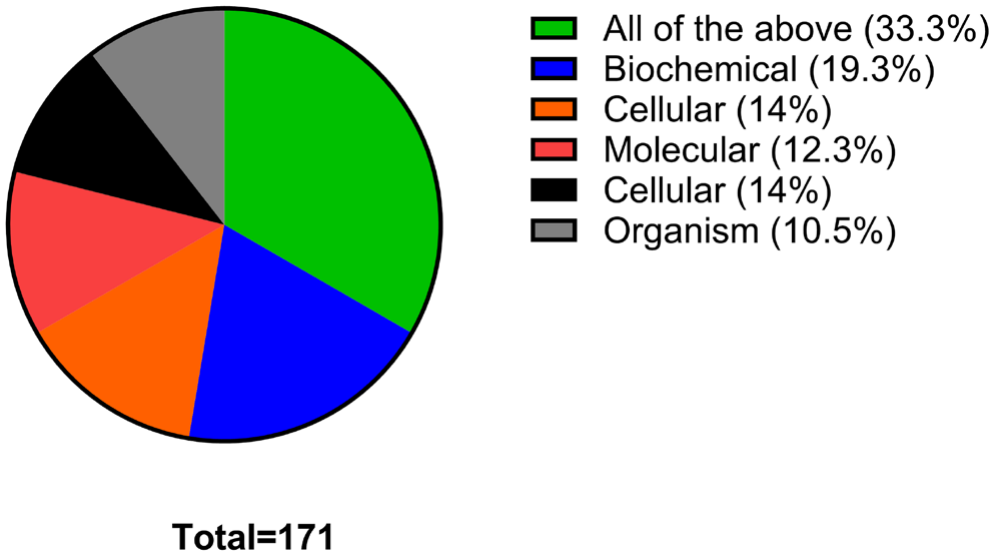
Pharmacologists investigate the action of drugs at which one or more level? Students were expected to indicate all answers that applied.

**FIGURE 7:**
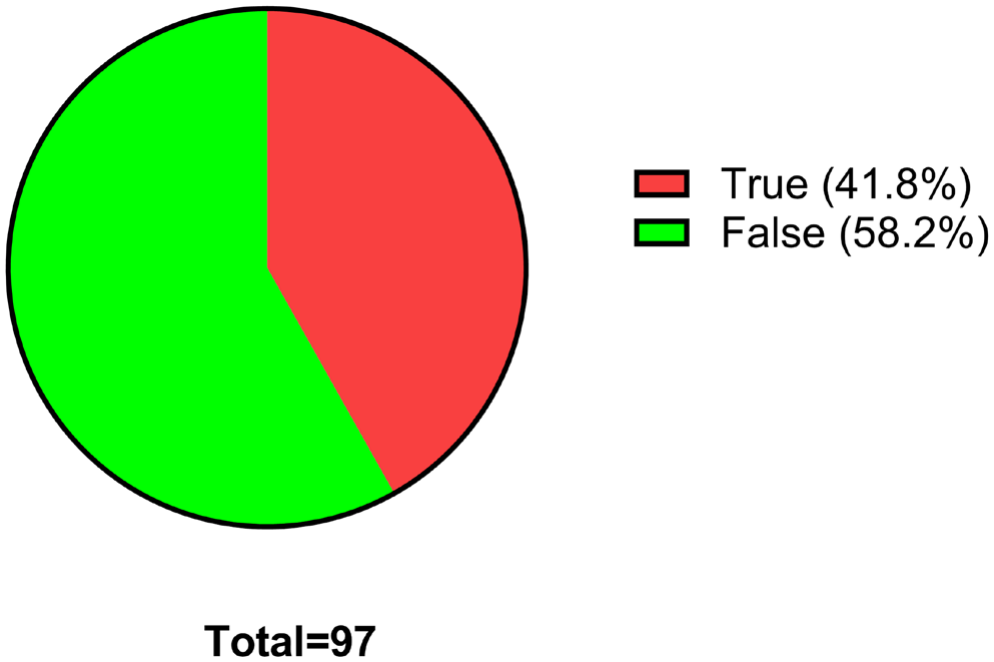
A degree in pharmacology enables graduates to work only as pharmacologists. Students were requested to provide a single response for this survey question.

In Question 5, students were asked to select from a list of statements which ones best describe what they thought pharmacologists do. The most popular statements were to develop new medicines with better therapeutic effects and fewer side effects (24.8%), understand more about how drugs work (21.2%) and develop medicines to treat diseases that have no current treatments (17.6%), which are accurately attributable to pharmacology (Figure 5). However, students are not aware that pharmacological research helps to understand more about how the body works, which was ranked only at 8.8%. Some students seemed to think that pharmacologists are responsible for dispensing medicines at pharmacies/hospitals (8.6%). In order to gain more insight into how much students know about pharmacology and pharmacological research approaches, students were asked to select at which organizational level pharmacologists study the action of drugs on the body. As seen in Figure 6, the options given, including the students' rankings in brackets, were as follows: molecular (12.3%), organ (10.5%), cellular (14%), biochemical (19.3%), organism (10.5%) and all of the above (33.3%). Despite the last answer, which is the correct one, being the most selected response, students decided to also select the other options. This highlights the level of confusion and somewhat limited knowledge among the students on pharmacology. To further assess the depth of knowledge that students have on pharmacology, in Question 7, students were asked if they thought that a degree in pharmacology enables graduates to only work as pharmacologists. Over 58% of the students selected false, indicating that pharmacology graduates can work in other disciplines and fields, while the remaining 41.8% believed that pharmacology graduates can only work as pharmacologists (Figure 7). This outcome is close to a 50:50 breakdown, which further indicates that many students are poorly acquainted with the careers of pharmacology graduates.

### 3.3 The Outcomes From Survey Questions Related to the Students' Opinion in Relation to Pharmacology Education in the Secondary Education Curriculum

**FIGURE 8:**
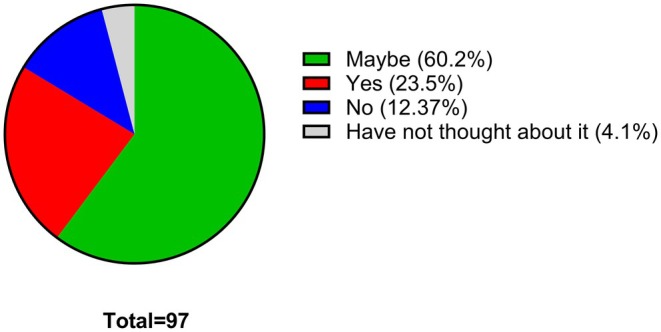
If you were taught about pharmacology as part of your current studies, would you consider studying it at university? Students were requested to provide a single response for this survey question.

**FIGURE 9:**
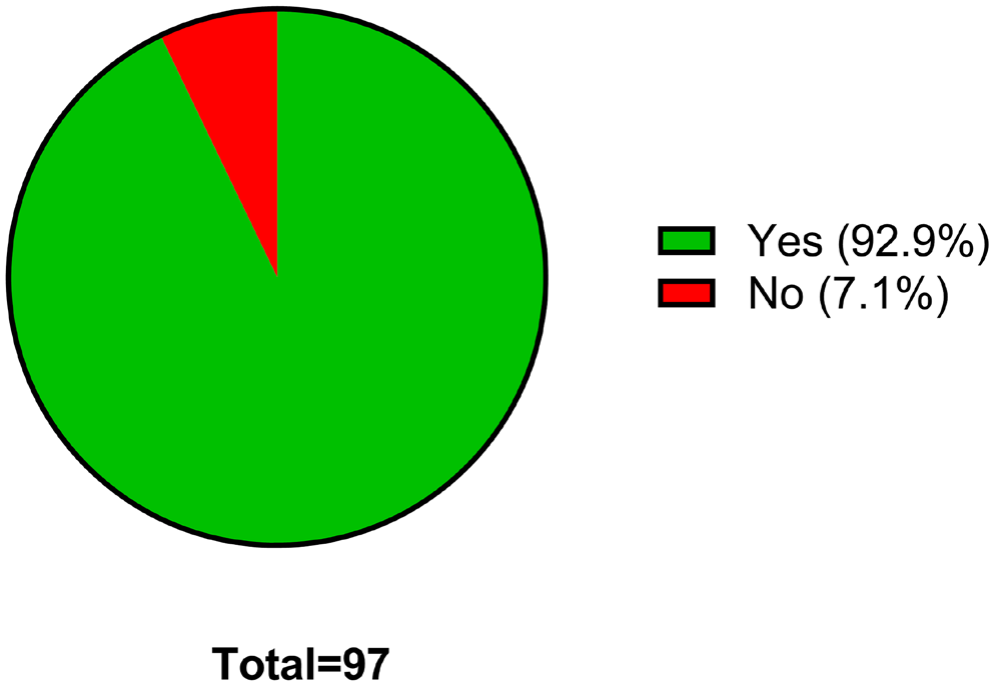
Do you think pharmacology should be introduced to secondary or further education curriculum? Students were requested to provide a single response for this survey question.

As described in the methodology section, after the students responded to Questions 1–7 and after being given a good overall introduction to pharmacology through the “Studying Pharmacology at University” video, the student were given the second part of the survey. In this part of the survey the students were asked about their opinion in relation to being taught about pharmacology as part of their FE curriculum and whether they would consider studying pharmacology at university. For the latter question (Question 8), 60.2% of the students stated that they might consider studying pharmacology at university if they had received a good introduction to it as part of their FE curriculum with a further 23.5% giving a clear and definitive yes to this question (Figure 8). In the final question for this survey, students were asked about whether they thought an introduction to pharmacology should constitutive a core part of their FE curriculum (Question 9). An overwhelming majority (92.2%) have stated that they would like to see an introduction to pharmacology in their secondary education curriculum (Figure 9).

## 4 Discussion

It is interesting and also unexpected that the vast majority of survey participants have reported that they have heard about pharmacology (almost 88%). Although this particular outcome was not expected due to the limited mention of pharmacology in the further education curriculum, in today's world of easy access to information through the internet and social media, then perhaps this outcome becomes less surprising. The inclusion of pharmacology education in the pre‐university biology and chemistry education will take this figure to 100%, which will mean that students will make more informed decisions about their career choices when applying for university courses and when deciding to study for a degree in pharmacology or something else. However, the depth of knowledge on pharmacology will also be important in this respect, as the other survey questions reveal (discussed below). It does appear that career advisors and teachers at participating educational institutions are playing an important role in informing students about pharmacology, and combined with the students' own research through the UCAS website, they constitute 46.4% of the sources through which students have heard about pharmacology. It is interesting and intriguing that 18.7% of the students reported that they have heard about pharmacology from friends or family members. It would have been useful to interrogate this aspect further, along with students reporting that they have heard about pharmacology through careers events (11.4%), documentaries (6%) and general press (6%).

Questions 3–6 address the level of knowledge that students have about pharmacology. Only 9.8% of the students associated pharmacology with biology, while 15.8% associated pharmacology with chemistry. Pharmacology has a strong biological element, which students do not seem to be aware of as revealed by these results. A report published in 2017 from a study entitled “Pharmacology Education and Education Pathway” commissioned by the British Pharmacological Society revealed that despite the widespread growth of pharmacology courses at university, there is a much higher percentage of pharmacology graduates; 30% higher than the number of course acceptances 3–4 years previously indicative of the fact that pharmacology courses have a high proportion of students transferring from other courses. This particular statistic points to the fact that students gain sufficient knowledge on what a degree and career in pharmacology entail after joining university [9]. The same study identified that there is a steady increase in recruitment of students into pharmacology courses from the highest socioeconomic bracket at the expense of students from lower middle class of socioeconomic groups. This is a matter that requires particular attention by the community of pharmacology educators in collaboration with the Department of Education. This study provided useful insights on the perceptions that pre‐university students have about pharmacology. It would also be interesting to conduct a larger study covering a wider geographical distribution and to survey more students from more colleges.

The study by Kwiek et al. [10] seems to be the only piece of research that has looked into the impact of pharmacology education among pre‐university students. This study addressed the issue of low attainment in biology and chemistry among high school students in the USA, and the authors suggest that the introduction of pharmacology education could help to improve the students' attainment by making the subjects more interesting and relevant. Initially, students were asked what they would be interested in learning about within the areas of biological and chemical sciences. Among the many responses received, the students highlighted their interest in learning about therapeutics and recreational drugs, as well as biological and chemical weapons. On that basis, a Pharmacology Education Partnership was formed with Duke University, North Carolina, and high schools across the United States, which included the development of modules with pharmacology content. The modules taught covered pharmacology concepts with relevance to both biology and chemistry, which were given eye‐catching titles including Acids, Bases and Cocaine Addicts, Drug Testing: A Hair‐Brained Idea, How Drugs Kill Neurons: It's Radical!, Military Pharmacology: It Takes Nerves, Why Do Plants Make Drugs for Humans?, and Steroids and Athletes: Genes Work Overtime. Most teachers reported that the incorporation of the above topics into the biology and chemistry lessons has helped to improve the students' performance in these topics. In fact, there was a positive correlation in students' performance with the number of topics covered out of the six above topics.

Research on the impact of pharmacology education at the pre‐university level is limited. However, several studies have been conducted with the aim of determining the best teaching strategies for pharmacology education to nursing and medical students. For example, it was shown by Thomas and Schuessier [11] that in order to better engage nursing students with pharmacology education, a topic that students tended to underperform in, the introduction of case studies, games, and in some cases humor was beneficial. Banning [12], also highlighting issues related to pharmacology education for nursing students, has put forward a number of proposals to help improve pre‐registration nursing students performance with an emphasis on relevant skills development.

We propose that the introduction of pharmacology education in the further education curriculum will improve the students' academic performance in biology and chemistry subjects, as clearly demonstrated by Kwiek et al. [10] and help the students to appreciate the relevance and significance of the biological and chemical concepts currently taught within these subjects. In addition, the introduction of pharmacology education at this early stage will help the students to make more informed decisions when applying for relevant university courses and to also gain some relevant insights on pharmacology that will be helpful when studying for clinically relevant courses such as medicines, nursing, and pharmacy, which have strong pharmacology elements that students, generally speaking, struggle with [13, 14, 15, 16, 17, 18, 19, 20].

## Author Contributions

**Cara Camilla Chantry:** data curation, investigation, methodology. **Elisa Salinas Pérez:** data curation, investigation, methodology. **Sahil Seyal:** data curation, investigation, methodology. **Saarah Mohammed:** data curation, investigation. **Devyani Deshai:** data curation, investigation, methodology. **Kristine Ofori:** data curation, investigation, methodology. **Muhummed Awan:** data curation, investigation. **Rayna Haque:** data curation, investigation. **Florence Olivia Mehtar:** data curation, investigation. **Samir S. Ayoub:** data curation, formal analysis, investigation, methodology, supervision, writing – original draft, writing – review and editing.

## Conflicts of Interest

The authors declare no conflicts of interest.

## Supporting information


**Appendix S1:** prp270157‐sup‐0001‐AppendixS1.docx.

**Appendix S2:** prp270157‐sup‐0002‐AppendixS2.docx.

## Data Availability

Data will be available upon request.
