# Supplementary material for: The Perception of Pharmacology Among College Students: An East London Perspective
Source: Pharmacol Res Perspect. 2025 Jul 28;13(4):e70157. doi: 10.1002/prp2.70157 (PMC12301629; doi:10.1002/prp2.70157)
Supplement: Supplementary file 1 — Appendix S1: prp270157‐sup‐0001‐AppendixS1.docx. [file PRP2-13-e70157-s002.docx]

**CONSENT FORM**

Programme of Study: BSc Pharmacology

Title of Project: The Perception of Pharmacology among secondary and further education students and educators

Project Supervisor: Dr Samir Ayoub

I confirm that I have read the information sheet for the above study and I have been given a copy to keep.

I understand what the study is about and I have had the opportunity to discuss with the researcher and ask questions about the study.

The procedures involved have been explained to me. I know what my part will be in the study and how the study may affect me.

I understand that my involvement in this study and particular data from this research will remain strictly confidential. Only researchers involved in the study will have access to the data.

It has been explained to me what will happen to the data once the study has been completed.

I understand that I have the right to stop taking part in the study at any time and I am not obliged to give any reason.

I know that if I do withdraw, it will not disadvantage me.

I know who to contact if I have any questions/concerns about my participation and I have their contact details.

I fully and freely consent to participate in the study.

Please tick to agree

Participant’s name (BLOCK CAPITALS)

Participant’s signature:

Date:

Researcher’s name (BLOCK CAPITALS)

Researcher’s signature:
Date:

**PARENTAL CONSENT FORM**

Programme of Study: BSc Pharmacology

Title of Project: The Perception of Pharmacology among secondary and further education students and educators

Project Supervisor: Dr Samir Ayoub

**Please tick to agree**

1. I confirm that I have read the information sheet for the above study and I have been given a copy to keep.
2. I understand what the study is about and I have had the opportunity to discuss with the researcher and ask questions about the study.
3. The procedures involved have been explained to me. I know what my child’s part will be in the study and how the study may affect him/her.
4. I understand that my child’s involvement in this study and particular data from this research will remain strictly confidential. Only researchers involved in the study will have access to the data.
5. It has been explained to me what will happen to the data once the study has been completed.
6. I understand that I have the right to stop my child taking part in the study at any time and I am not obliged to give any reason.
7. I know that if I do withdraw my child, it will not disadvantage me or him/her.
8. I know who to contact if I have any questions/concerns about my child’s participation and I have their contact details.
9. I fully and freely consent to my child participating in the study.

Parent’s name **(BLOCK CAPITALS) _______________________________**

Parent’s signature: **________________________________ Date: _________________**

Researcher’s name **(BLOCK CAPITALS) ____________________________**

Researcher’s signature: ____________________________ **Date: _________________**
