# Supplementary material for: The Perception of Pharmacology Among College Students: An East London Perspective
Source: Pharmacol Res Perspect. 2025 Jul 28;13(4):e70157. doi: 10.1002/prp2.70157 (PMC12301629; doi:10.1002/prp2.70157)
Supplement: Supplementary file 2 — Appendix S2: prp270157‐sup‐0002‐AppendixS2.docx. [file PRP2-13-e70157-s001.docx]

**INFORMATION SHEET FOR PARTICIPANTS**

**Form for Students**

Programme of Study: BSc Pharmacology

Title of Project: The Perception of Pharmacology among secondary and further education students and educators

Dear Participant,

You are being invited to take part in a research study. Before you decide whether to participate, it is important for you to understand why the research is being done and what it will involve. Please take time to read the following information carefully and ask us if there is anything that is not clear or if you would like more information. Take time to decide whether or not you wish to take part.

**What is the purpose of the study?**

- To gain an insight on the perception of pharmacology among years 8-13 students and their teachers

**What will I have to do if I take part?**

- You will be expected to complete a 2-part survey. Between parts 1 and 2 of the survey you will be shown a video giving you an overview on pharmacology. Completion of this survey will take a maximum of 30 minutes, mostly less than that (20 minutes) and this will take place in your usual classroom at a time that your teachers have identified as appropriate.

**What are the possible advantages of taking part?**

- To gain an insight on the perception of years 8-13 students and their teachers on pharmacology with the aim of using these data as evidence to present to policy-makers to consider the introduction of pharmacology education at some point in the secondary and FE curriculum.

**What are the possible disadvantages or risks of taking part?**

- No disadvantages or risks are associated with this study.

**Do I have to take part?**

You are under no obligation to participate in this study. If you do decide to take part, you are free to withdraw at any time without giving a reason. If you do not take part or withdraw from the study at a later date, it will not disadvantage you. Once data collection has been completed and the study has gone into the data analysis phase, you can withdraw up until 30^th^ November 2019.

**What will happen to the information?**

Your participation in this study and all information collected will be kept strictly confidential. Where necessary, information collected will be coded so that you cannot be recognised from it. **No personal information will be collected at any part of the study** The results of this study will be reported as part of my degree programme and may be further disseminated for scientific benefit. The results will be available to you on request.

**Who should I contact for further information or if I have any problems/concerns?**

Study supervisor: Dr Samir Ayoub (email: [s.s.ayoub@uel.ac.uk](mailto:s.s.ayoub@uel.ac.uk), Tel 0208 223 4077)

In the event that you have any concerns regarding the conduct of the study, please contact: Catherine Hitchens, Research Integrity and Ethics Manager, EB 1.43 Graduate School, Docklands Campus, London, E16 2RD.  Telephone 0208 223 6683.  Email: [researchethics@uel.ac.uk](mailto:researchethics@uel.ac.uk).
